# Accuracy in WiFi Access Point Position Estimation Using Round Trip Time

**DOI:** 10.3390/s21113828

**Published:** 2021-06-01

**Authors:** Miquel Garcia-Fernandez, Isaac Hoyas-Ester, Alex Lopez-Cruces, Malgorzata Siutkowska, Xavier Banqué-Casanovas

**Affiliations:** Rokubun S.L., 08018 Barcelona, Spain; isaac.hoyas.ester@rokubun.cat (I.H.-E.); alex.lopez@rokubun.cat (A.L.-C.); malgorzata.siutkowska@rokubun.cat (M.S.); xavier.banque@rokubun.cat (X.B.-C.)

**Keywords:** WiFi, Round Trip Time, fine time measurements, Access Points, GNSS, navigation

## Abstract

WiFi Round Trip Time (RTT) unlocks meter level accuracies in user terminal positions where no other navigation systems, such as Global Navigation Satellite Systems (GNSS), are able to (e.g., indoors). However, little has been done so far to obtain a scalable and automated system that computes the position of the WiFi Access Points (WAP) using RTT and that is able to estimate, in addition to the position, the hardware biases that offset the WiFi ranging measurements. These biases have a direct impact on the ultimate position accuracy of the terminals. This work proposes a method in which the computation of the WiFi Access Points positions and hardware biases (i.e., products) can be estimated based on the ranges and position fixes provided by user terminals (i.e., *inverse positioning*) and details how this can be improved if raw GNSS measurements (pseudoranges and carrier phase) are also available in the terminal. The data setup used to obtain a performance assessment was configured in a benign scenario (open sky with no obstructions) in order to obtain an upper boundary on the positioning error that can be achieved with the proposed method. Under these conditions, accuracies better than 1.5 m were achieved for the WAP position and hardware bias. The proposed method is suitable to be implemented in an automated manner, without having to rely on dedicated campaigns to survey 802.11mc-compliant WAPs. This paper offers a technique to automatically estimate both mild-indoor WAP products (where terminals have both Wi-Fi RTT and GNSS coverage) and deep-indoor WAP (with no GNSS coverage where the terminals obtain their position exclusively from previously estimated mild-indoor WAPs).

## 1. Introduction

The WIFI 802.11mc protocol ([1]) allows a device to measure the distance to a WiFi Access Point (WAP) in a bi-directional communication process. The user terminal *initiates* this process, and the WAP *responds* to the query, which eventually results in an estimation of the distance through the Round Trip Time (RTT, details on the protocol can be found in, e.g., [2]). As of today, the amount of devices supporting this protocol is scarce, limited to few router brands that are able to both *announce* support for the 802.11mc protocol and *respond* to RTT queries (examples are Compulab WILD and Google WiFi routers as well as other mesh network routers). On the *initiator* side, to the authors’ best knowledge, only Google Pixel and other high end smartphones support this feature ([3]), albeit other open platforms based on Intel chipsets have also been proposed (see, e.g., [4,5]).

This technology promises a breakthrough in indoor navigation, particularly in a smart city environment where smartphones are ubiquitous and applications based on this technology can enhance Location-Based Service applications ([6]). While the benefit of this technique will be mostly evidenced in indoor environments, using RTT-based WiFi positioning could potentially be used to assist GNSS in areas with a lack of visibility (i.e., urban canyons) or to reduce the time-to-first-fix by providing a meter-level a priori position.

In addition, RTT-based positioning could serve as an alternate position provider in GNSS denied or compromised (i.e., spoofed) environments. Indeed, the measurement accuracy of the RTT ranges is on the order of tens of decimeters (see, for instance, [7]), which certainly allows meter-level accuracy (as shown in [8,9,10,11,12]). However, besides environmental error sources, such as multipath, RTT ranges are usually offset due to biases introduced by the WAP hardware ([13]).

Despite this potential breakthrough, there is a clear limitation of the 802.11mc protocol due to its bi-directional nature, as opposed to other navigation systems, such as Global Navigation Satellite Systems (GNSS), that are unidirectional (i.e., user terminals only receive and do not transmit information to the satellites). The fact that an *initiator* has to start a dedicated communication with the WAP limits the scalability of systems based on WiFi RTT. Some works propose methodologies to synchronize a set of WAPs so that the complete system is more GNSS-like (i.e., unidirectional, see [2,14]), which would enable a fully scalable solution.

Thus far, most works on WiFi RTT deal with the positioning of the user terminal using WiFi RTT or a hybridized strategy with ranging GNSS ([15]); however, WAP positioning remains a topic to be fully investigated. An accurate estimation of the WAP position will impact the final accuracy in the user terminal, and thus lowering the WAP positioning error is critical. In previous works, WAP positions were usually obtained by survey campaigns using geodetic grade GNSS receivers (see, for instance, [16]).

However, this solution is not practical for operational systems, and thus an automated methodology should be developed. This work intends to provide a solution based on the fact that raw GNSS measurements have been available in Android devices since 2016 ([17]) which unlocks the possibility of having sub-metric accuracy in smartphones ([18,19,20]). Therefore, geotagging the RTT measurements with a more accurate user terminal position has, in turn, the potential to better locate the position of WAPs.

In the following paper, we intend to provide a methodology to estimate the WAP position and hardware bias as well as to improve these estimates by means of exploiting the better accuracy offered by processing the raw GNSS measurements. The paper starts with a description of the data processing model and is followed by the Results section, describing the data campaign executed to test the model and the quality assessment of the estimation of WAP position and hardware bias as well as its impact in the terminal location. The paper is concluded with the Discussion section, which contains our conclusions of the work.

## 2. Data Processing Model

In a similar way as in other range-based navigation systems, such as GNSS (see, for instance, Section 6.1.1 of [21]), the basic measurement to compute a receiver (rx) position based on the known coordinates of a set of transmitters (tx) is the range between the receiver and these transmitters. This geometric distance (ρ) can be computed as:

(1) $$\rho =\sqrt{{\left(x_{tx}-x_{rx}\right)}^{2}+{\left(y_{tx}-y_{rx}\right)}^{2}+{\left(z_{tx}-z_{rx}\right)}^{2}}$$

which can be linearized using a first order Taylor expansion, which requires an a priori knowledge of the element to be geolocated. Albeit this linearization can yield to biases in the estimated state, specially if the a priori is too coarse, it is needed when estimation strategies based on Kalman filtering are used [22]. The linearization would yield the following expression:

(2) $$\rho ≃\frac{x_{tx}-x_{rx,0}}{\rho_{0}}\cdot \Delta x+\frac{y_{tx}-y_{rx,0}}{\rho_{0}}\cdot \Delta y+\frac{z_{tx}-z_{rx,0}}{\rho_{0}}\cdot \Delta z$$

where $\rho_{0}$ is the distance between the WiFi Access Points (WAP) (i.e., *transmitters*) and the a priori position of the user terminal to geolocate (*receivers*): ${\vec{r}}_{rx,0}=\left(x_{rx,0},y_{rx,0},z_{rx,0}\right)$. A possible method to compute the approximate a priori position of the user terminal could be by means of the Bancroft method (see, for instance, Appendix D of [23]).

In the ideal case (i.e., no biases and no measurement errors), if the user terminal measures the RTT from at least three WAPs, the position delta relative to the a priori ($\Delta \vec{r}=(\Delta x,\Delta y,\Delta z$)) can be solved by a simple Root Mean Square solution. While WiFi RTT ranging is a two-way communication system (as opposed to GNSS), and therefore no receiver clock has to be estimated, previous works showed that the RTT measurements were affected by an offset or bias ([13]). Therefore, the WiFi RTT range measurement ($\rho_{RTT}$) is better modeled using the following expression:

(3) $$\rho_{RTT}=p_{x}\cdot \Delta_{x}+p_{y}\cdot \Delta_{y}+p_{z}\cdot \Delta_{z}+b_{hw}+\varepsilon_{\sigma }$$

where $p_{⋄}$ is the *partial* of the ⋄ component, defined as:

(4) $$p_{⋄}=\frac{{⋄}_{tx}-{⋄}_{rx,0}}{\rho_{0}},$$

where $b_{hw}$ is the hardware bias (offset) introduced by the Access Point, as pointed out in [13] and is expressed in meters. Finally, $\varepsilon_{\sigma }$ is the modeled thermal (Gaussian) noise of the measurement (with standard deviation σ). The user terminal position can be, therefore, estimated by solving the following linear system of *N* equations (one equation per each WAP for which there is a RTT measurement):

(5) $$\left(\begin{array}{c} \rho_{RTT}^{1}-\rho_{RTT,c}^{1}\\ \rho_{RTT}^{2}-\rho_{RTT,c}^{2}\\ \vdots \\ \rho_{RTT}^{N}-\rho_{RTT,c}^{N} \end{array}\right)=\left(\begin{array}{ccc} -p_{x}^{1} & -p_{y}^{1} & -p_{z}^{1}\\ -p_{x}^{2} & -p_{y}^{2} & -p_{z}^{2}\\ \; & \vdots & \\ -p_{x}^{N} & -p_{y}^{N} & -p_{z}^{N} \end{array}\right)\cdot \left(\begin{array}{c} \Delta x\\ \Delta y\\ \Delta z \end{array}\right)$$

where the *prefit* residuals for the i−th WAP ($\rho_{RTT}^{i}$ - $\rho_{RTT,c}^{i}$) are the measured RTT range minus the computed RTT range (using the model proposed in Equation (3)), and $p_{⋄}^{i}$ is the partial for the ⋄ component and for the i−th WAP.

As can be seen, to compute the user terminal, not only the WAP position but also the bias ($b_{hw}$) is needed. Not adding the bias into the navigation equations will increase the error of the estimated positions.

To solve the *inverse positioning* problem and obtain both the WAP location and bias (instead of the user terminal position), the linear system shown in Equation (5) needs to be slightly reformulated as follows:

(6) $$\left(\begin{array}{c} \rho_{RTT,1}-\rho_{RTT,c,1}\\ \rho_{RTT,2}-\rho_{RTT,c,2}\\ \vdots \\ \rho_{RTT,M}-\rho_{RTT,c,M} \end{array}\right)=\left(\begin{array}{cccc} p_{x,1} & p_{y,1} & p_{z,1} & 1\\ p_{x,2} & p_{y,2} & p_{z,2} & 1\\ \; & \vdots & \; & \\ p_{x,N} & p_{y,M} & p_{z,M} & 1 \end{array}\right)\cdot \left(\begin{array}{c} \Delta x_{tx}\\ \Delta y_{tx}\\ \Delta z_{tx}\\ b_{hw} \end{array}\right)$$

Instead of *N* equations in this case we have *M* measurements collected by the smartphone. These *M* measurements can be taken from a single terminal at different geographical locations within the WAP area coverage or by simultaneous terminals under the WAP coverage at different locations and epochs. The combination of measurements from different terminals, locations and epochs can be done because the WAP hardware bias ($b_{hw}$) is relatively stable over time (as noticed by the authors as well as in [13]); therefore, measurements over different epochs can be processed in the same batch. This is slightly different than other navigation systems, such as GNSS, where the receiver clock biases depend over time, and thus measurements over different epochs have to be processed in different batches.

Clearly, the data processing model will not only need the RTT measurements as the basic observable but also the position at which this measurement was taken (i.e., the user terminal location). In smartphones, when using terminals such as Google Pixel 4, the RTT measurements can be geotagged with the position fixes provided by the Android Location Service. However, it is important to note that the error in the terminal position will directly impact the accuracy with which the WAP position can be estimated.

To improve the terminal location, other positioning algorithms can be used as shown later in this paper. Examples of such techniques include Precise Point Positioning (PPP, which uses code and carrier GNSS measurements as well as precise orbits and clocks) and Post-Processing Kinematics (PPK, which uses differential techniques with nearby GNSS reference stations). These techniques can be also applied in Android smartphones due to the fact that, since 2016, the Android API has granted access to the GNSS raw measurements to the developers (see [17,24]).

## 3. Data Campaign and Setup

**Figure 1:**
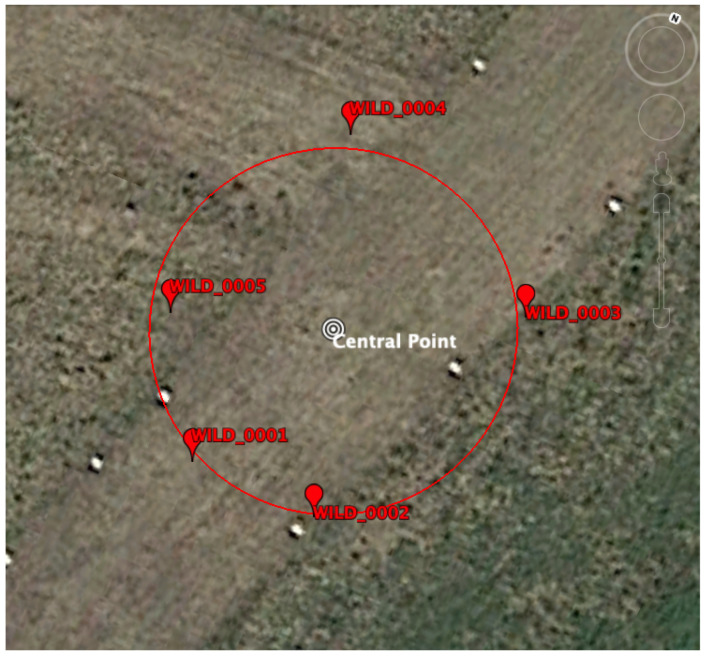
The five WiFi routers were placed around a circle of 10 m, centered at the Central Point (CP). The positions of the routers and CP where surveyed using a Septentrio AsteRx GNSS receiver and a Post Processing Kinematic (PPK) strategy.

In order to test the data processing model presented above and assess its best possible performance (upper boundary on the positioning error), a data campaign was carried out to collect RTT measurements in a controlled and benign setup (open sky and no obstructions). The hardware equipment used in this campaign corresponds to five WiFi Access routers from CompuLab (WILD), which were RTT compliant, and two Google Pixel 4 smartphones. The five WiFi routers (red) were placed in a concentric circle of 10 m (red circle) centered at the Central Point shown in Figure 1 as a white circle.

The positions of each Access Point were surveyed using a geodetic-grade GNSS receiver (Septentrio AsteRx with a PolaNt antenna) using a Post Processing Kinematic (PPK) strategy that allows achieving centimetric accuracy in the position estimates (see, for instance, [25,26]). These positions will be the truth reference to which the estimated Access Point locations will be compared to. The positions as well as the network identifiers (SSID) are collated in Table 1.

In addition to surveying the location of the Access Points, the Central point location was also measured using a geodetic-grade GNSS receiver. This allowed us to obtain an accurate distance between the Access Points and this central point, which is collated in Table 2

### Hardware Bias Estimation

As already pointed out in the data processing section and in [13], WiFi RTT measurements might have an offset that, if not modeled, will directly impact the positioning accuracy of the user terminals. Therefore, a service providing the WiFi Access Point (WAP) position should also provide an estimation of the WAPs $b_{hw}$ so that end-users can obtain the best possible WiFi-based positioning. This service should jointly estimate both the position as well as the hardware bias $b_{hw}$ of the WAP at the same time, through the solution of the linear system of equations represented in Equation (6).

This section attempts to assess the typical size of this hardware bias $b_{hw}$ in a controlled environment using an independent method that is different from the proposed inverse positioning technique—the average difference between the observed RTT ranges and the true measured ranges (the geometrical distance between the terminal and the Access Point). This will be used later when comparing the results obtained with the inverse positioning technique.

To achieve this, the data set described in the previous section was used: the two smartphones were placed at the central point (CP). The relative distances were estimated with centimetric accuracy as the difference between the surveyed positions of the CP and the WAP location. Both the CP and WAP locations were estimated using a Post-Processed Kinematics (PPK) strategy (with centimeter-level accuracy, see, for instance, [26]). These relative distances are collated in Table 2.

**Figure 2:**
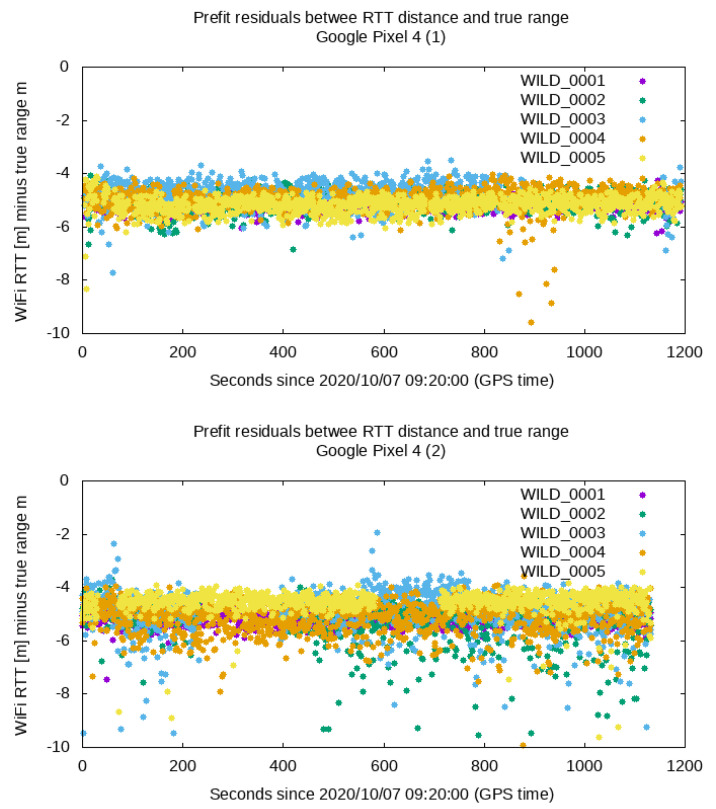
Prefit residuals of the RTT measurements (observed by the smartphones placed at CP minus the true distance).

In order to estimate the biases, the *prefit residuals* of the WiFi RTT measurements collected by the two smartphones at the CP were obtained (i.e., the observed RTT range measurements minus the true distance to the router). In the ideal case where no $h_{bw}$ is present, the resulting prefit residuals would be 0. In reality, a bias was evidenced, as shown in Figure 2. The prefit residuals were clearly biased by an amount that is on the meter level (ca. −5 m), consistent with magnitudes observed in other works ([13]). A simple averaging of the prefit residuals for each router yielded the biases, which are collated in Table 3.

An important result that can be obtained from this analysis is the standard deviation of these prefit residuals, which provides an indication of the the thermal noise for the RTT measurements. As it can be seen, this was comprised between 20 and 70 cm and compatible with the measurement error reported by Android devices (by the field DistanceStdDevMm, see [27]) and already reported in previous works ([28]). No evident pattern was observed between the hardware bias and the environment of the smartphone. However, the hardware bias did seem to depend on the frequency at which the RTT measurements were collected (i.e., 2.4 or 5 GHz), according to further data samples collected after this test campaign.

## 4. Results

### 4.1. Wifi Access Point Positioning

**Figure 3:**
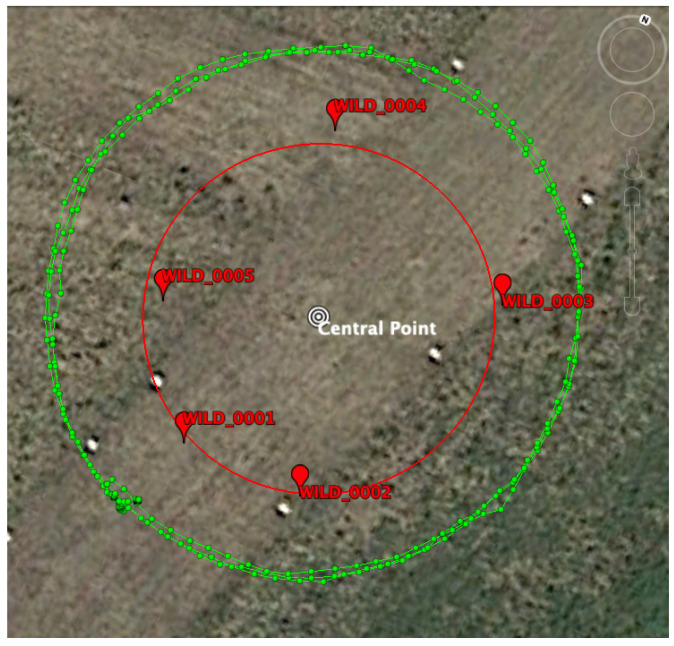
The smartphones followed various circle of a ca. 15-meter radius that contained the whole setup shown in Figure 1.

This section contains the results of the WAP positioning estimate (as well as the hardware biases) using the processing model described above. RTT measurements from the two Google Pixel 4 smartphones were collected with a tailored app while walking around various circles outside the setup, as shown by the green line in Figure 3.

As can be seen, the environment was open sky without obstructions. Therefore this can be considered as a benign scenario and a measure of the best possible accuracy that can be obtained using WiFi RTT measurements collected with a smartphone. A more realistic scenario will likely yield worse results due to environmental errors, such as multipath (nearby walls and obstructions), signal blockage (user holding the smartphone), and higher dynamics (turns and accelerations, *…*).

As mentioned before, in order to perform the *inverse positioning* and estimate the positions of the WAP, the RTT measurements need to have an associated position. For this work, two positions were considered: the *Android position*, where the position was obtained from the user terminal itself (Android Location Service), and the *Jason Position*, where the position was estimated using PPK computed with the GNSS measurements from the user terminal.

Due to the feature that allows extracting the GNSS raw measurements from smartphones, in particular, the carrier phase (e.g., [29]), accessing sub-meter accuracy on those devices is possible by applying differential techniques, such as PPK (see, for instance, [30]). The app developed as a data grabber of RTT measurements for this work contains marks for synchronisation with the GPS time scale. This feature is needed to retag those measurements with a sub-meter accuracy. To do this, the GNSS data gathered by the smartphones was uploaded to Rokubun’s Jason cloud GNSS service ([31]) to obtain a PPK solution. The resulting position estimates (timetagged with the GPS time scale) were used to interpolate the position at the epochs where the RTT measurements were taken.

To evaluate the accuracy of the proposed *inverse positioning* strategy to compute the WAP positions, the obtained positions were compared against the reference positions surveyed with the Septentrio AsteRx receiver (see Table 1). These basic metrics were considered as performance indicators:

- Position errors—essentially the horizontal and vertical errors. These errors can be computed using a tangential reference frame (East/North/Up): as is known, given a reference position and a 3D error vector, the East and North components are the projection of the error vector in the East and North directions, respectively, while the Up component is the projection of the error vector in the vertical direction. In the tangential reference frame, the vertical error ($\Delta_{vertical}$) is directly the Up component, while the horizontal deviation ($\Delta_{horizontal}$) is defined in Equation (7).
- Hardware bias errors—computed as deviation to the reference values described in the previous section (see Table 3).

(7) $$\Delta_{horizontal}=\sqrt{\Delta_{east}^{2}+\Delta_{north}^{2}}$$

The results for these metrics are collated in Table 4 and Table 5 for the two strategies to tag the RTT measurements described above (using *Android location* and retagging with Jason, respectively). The most noticeable difference was due to the improvement in the horizontal error when using the Jason service (to retag the RTT measurements with the PPK position estimates): when using *Android location*, errors larger than 2.5 m were obtained, while they were clearly reduced to less than 1.5 with the *Jason location*.

The vertical errors were, in general, larger than the horizontal ones, but this is because of the geometry distribution of the setup. As is known from other navigation systems, such as GNSS, the Dilution-Of-Precision (DOP) causes an error amplification when the geometry of the receivers and transmitters is not *diverse* (i.e., all observations aligned, see, for instance, Section 6.1.2 of [21]).

Due to the fact that, in the proposed setup, all transmitters and receivers were in the same plane, the vertical geometry was worse than the horizontal geometry. Better vertical accuracy could have been obtained if RTT measurements above the routers were available. The DOP is usually quantified using the geometry matrix of the observations, and the lower the value is, the less error amplification. In the proposed setup, horizontal DOP of around 3 were obtained, while the vertical DOP was about 50, indicating a high difference in the geometric diversity between these two components.

Concerning the hardware bias, in general, the resulting hardware biases were estimated within 1 to 1.5 m relative to the values provided in the previous section, and no substantial differences between both strategies were detected. However, these differences are within the same magnitude of the thermal noise error of the RTT measurements (see the last column of Table 3 and Figure 2).

### 4.2. Impact on the Terminal Accuracy

The ultimate goal of having the positions and hardware biases of the WAPs is to provide the best possible accuracy to user terminals using the RTT, particularly in areas with limited or no GNSS coverage (for instance in deep indoor environments). This section outlines the expected accuracy that can be achieved using the service based on the methodology proposed in this paper, also known as the WAP Location Service (WALS). This section is not intended to provide a full report on using WiFi RTT for user terminal navigation, as this has been widely covered in previous references (see, for instance, [9,10,15] and the references therein). Rather, we intend to gauge the impact of using different sets of WAP positions and biases in the position of the user terminals.

For this test, the two smartphones were placed at the Central Point. They collected Wifi RTT measurements for ca. 1 min and then were processed using Rokubun’s navigation filter using two sets of WAP coordinates and biases: (a) the ones obtained by surveying the position with the Septentrio GNSS receiver (*True position*) and (b) the ones obtained with the proposed service (*WALS position*).

**Figure 4:**
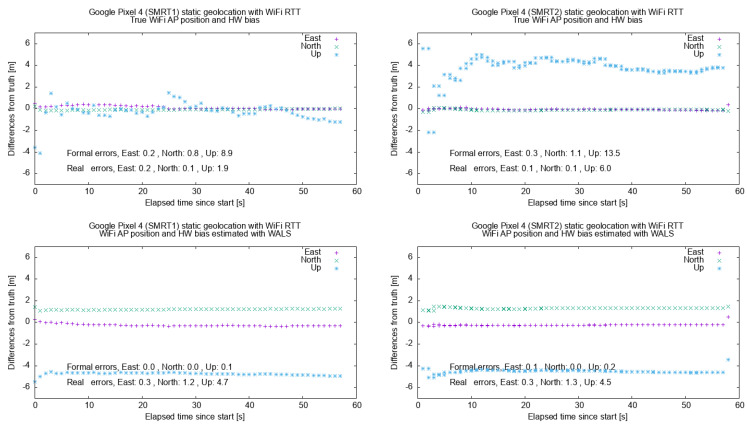
Result for the two Google Pixel smartphones (user terminal) in static positioning using the *true* WAP position and biases (**above**) and *WALS* WAP position biases (**bottom**).

The panels in Figure 4 show the East/North/Up deviation relative to the reference position of the Central Point. The figure summarizes the results for the two smartphones used in the test, using the true positions and hardware biases (upper panels) and using the ones delivered by WALS instead (lower panels). As expected, the differences (especially in the vertical dimension) increased due to the fact that the WAP position and hardware biases delivered by WALS had estimation errors in the meter level (as reported in the previous section). However, the horizontal error was within 2 m. The vertical component showed the larger deviation due to the worse DOP in the vertical dimension, which amplifies any measurement error of the RTT ranges.

**Figure 5:**
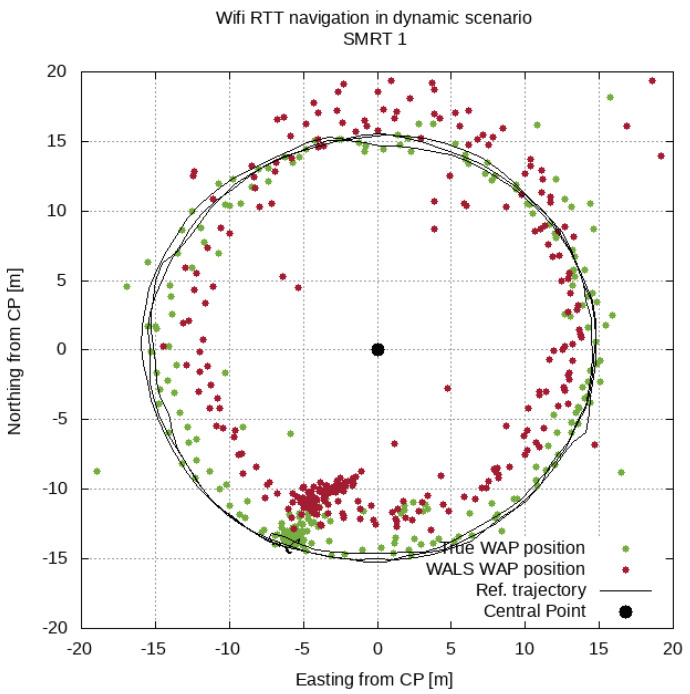
The results of the WiFi positioning in a dynamic environment using WAP positions obtained from a true reference or from the proposed methodology (WALS).

A more qualitative test can be performed in a dynamic test where the smartphones are moved around a circle with a constant radius of 15 m (similar to the green circle in Figure 3). The results for the horizontal dimension (easting and northing) are shown in Figure 5 for the two sets of WAP positions. As expected, the track at 15 m was better followed when more accurate WAP positions were provided (green track, using the true WAP positions) than when the positions provided by the proposed methodology (WALS) were used. However, the errors were still within the 2-m level.

## 5. Discussion

WiFi Round Trip Time measurements, enabled with the 802.11mc protocol, unlock the possibility of navigating with meter-level accuracy where there is no access to other navigation systems (e.g., GNSS does not work indoors). However, one key point to be able to perform this is to have accurate positions as well as the hardware (or system) biases of the WiFi Access Points (WAP). To the authors’ best knowledge, little attention has been paid so far to this problem, and previous works typically offered ad-hoc approaches, where the Access Points were surveyed with dedicated campaigns. This approach is not scalable, particularly considering the vast amount of potential RTT-capable WAPs that might appear in the future, and an automated system might be required to compute both the position as well as the biases of those WAPs.

We proposed an inverse positioning methodology by which user terminals provide the RTT ranges geotagged with the terminal position in order to estimate the position of the Access Points. Moreover, this estimation was improved using raw GNSS measurements (pseudoranges and carrier-phases) provided by the user-terminal in addition to the RTT ranges. In this case, differential GNSS techniques, such as PPK, can be used to improve the positions at which the RTT measurements were obtained causing a reduction in the error of the WAP position and hardware bias.

This process can be easily automated using an Application Programming Interface (API) of services, such as Rokubun’s Jason, which accepts GNSS data collected by smartphones and delivers the PPK position. Accuracies better than 1.5 m were achieved in the horizontal dimension, but the error in the vertical dimension increased due to a worse dilution-of-precision (DOP). In addition, The WAP biases can also be estimated with an accuracy of 1 to 1.5 m. Once the WAP positions and hardware biases are computed, they are stored in a database and served, via the API, to other users that need this data to compute their own positions in the GNSS denied environment (i.e., indoors).

It is unlikely that terminals sending data to the server to compute WAP products use these products to compute their own positions. Instead, terminals under the coverage of WAPs that are near windows in buildings (i.e., mild-indoors) will compute their locations using GNSS and then submit RTT measurements with these GNSS-based geotags.

The server will apply the inverse positioning technique to compute these mild-indoor WAP products with the accuracy level shown in this paper. At a later stage, other terminals could use these mild-indoor WAP products to compute their positions in locations without GNSS visibility (for instance well within the building, deep-indoors) and, potentially, geotag RTT measurements of other nearby WAP that are deep-indoors. These geotagged RTT measurements could be then sent to the server to compute the products of these deep-indoors WAPs, albeit with a potential decrease in accuracy.

**Figure 6:**
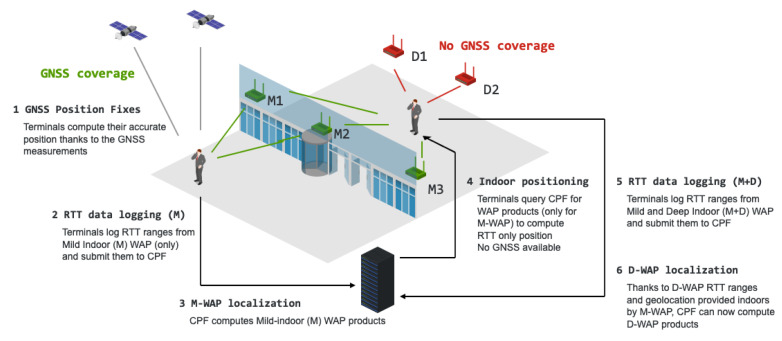
Process flow of the typical use case for the inverse positioning technique proposed in this paper.

A whole system level description of an architecure using the inverse positioning technique is shown in Figure 6. Terminals under coverage of WAPs that are near windows in buildings (i.e., mild-indoors) will compute its location using GNSS only (i.e., Wi-Fi RTT ranges may not be needed for this purpose at this stage). This user’s RTT measurements would be geotagged with this GNSS fix and then submit them to the server. The server will apply the inverse positioning technique to compute these mild-indoor WAP products with the accuracy level shown in the previous section. At a later stage, other terminals located indoors, with no GNSS visibility, could use these mild-indoor WAP products to compute its position. Eventually, the Wi-Fi only position fixes could be used to geotag RTT measurements of other nearby WAP that are deep-indoors. Note that these deep-indoor RTT measurements have not been used to compute the terminal position because, at this stage, the deep-indoor WAP products have not been yet computed and are not available to the user. Finally, these geotagged RTT measurements could be then sent to the server to compute the products of these deep-indoors WAPs.

The essential requirements in terms of hardware in order for this technique to be applicable is that terminals and WAP support Wi-Fi 802.11mc. A list of devices supporting this protocol is listed in [3]. Regarding the Access Points, besides the one listed in this reference, modern commercial mesh systems such as Netgear Orb, Amazon Eero or Linksys Velop do support also this protocol.

In the future, additional lines for future work include an analysis of the hardware bias stability for the same device in subsequent power cycles. Moreover, more realistic studies involving indoor environments should be considered to assess the real impact of obstructions and also to assess the extent to which WAP with no outdoor visibility (such as the ones placed in deep indoor environments) can be estimated exclusively with other WAP for which their positions and hardware biases could be successfully estimated with the proposed technique (i.e., WAP near windows or mild indoors).

## 6. Patents

As a result of the methodology described in this work, the following patent is currently under evaluation in the European Patent Office (EPO): GEOLOCATING WIRELESS ACCESS POINTS (Application number EP20194450.1 filed on 3 September 2020).

## Figures and Tables

**Table 1:** Reference position (ETRS89 reference frame) of the Wifi Access Points, surveyed with a Septentrio AsteRx GNSS receiver.

SSID	Longitude [deg]	Latitude [deg]	Height [m]
CP	2.1640443	41.8091309	931.137
WILD_0001	2.1639939	41.8090443	930.374
WILD_0002	2.1640822	41.8090430	930.518
WILD_0003	2.1641564	41.8091737	930.797
WILD_0004	2.1640039	41.8092222	930.690
WILD_0005	2.1639418	41.8091072	930.463

**Table 2:** Geometric distances between the setup Central Point (CP) and each of the routers considered in the data campaign.

SSID	Distance to CP [meters]
WILD_0001	10.5
WILD_0002	10.3
WILD_0003	10.3
WILD_0004	10.7
WILD_0005	8.9

**Table 3:** The estimated hardware biases (in meters) using the true positions of the Access Points and smartphone positions (surveyed with a Septentrio AsteRx GNSS recever). Both the estimated hardware bias as well as the precision of this estimation is shown in the table for each router.

SSID	$b_{\mathrm{hw}}$ [meters]	$\sigma_{\mathrm{prefit}}$ [meters]
WILD_0001	−5.124	0.22
WILD_0002	−4.933	0.67
WILD_0003	−4.609	0.67
WILD_0004	−4.880	0.59
WILD_0005	−5.018	0.49

**Table 4:** Results with RTT measurements tagged with the *Android Position*. Horizontal and vertical position deviations relative to the reference WAP positions and computed WAP hardware bias.

SSID	$\Delta_{\mathrm{horizontal}}$ [meters]	$\Delta_{\mathrm{vertical}}$ [meters]	$b_{\mathrm{hw}}$ [meters]
WILD_0001	2.6	3.4	−4.2
WILD_0002	2.8	−2.8	−3.4
WILD_0003	1.1	−0.9	−2.5
WILD_0004	0.9	−3.3	−2.5
WILD_0005	2.7	8.0	−3.6

**Table 5:** Results with RTT measurements tagged with the *Jason Position*. Horizontal and vertical position deviations relative to the reference WAP positions and computed WAP hardware bias.

SSID	$\Delta_{\mathrm{horizontal}}$ [meters]	$\Delta_{\mathrm{vertical}}$ [meters]	$b_{\mathrm{hw}}$ [meters]
WILD_0001	1.3	0.7	−4.3
WILD_0002	2.0	2.6	−3.5
WILD_0003	0.4	2.5	−2.7
WILD_0004	0.9	5.6	−2.2
WILD_0005	1.5	−1.2	−3.8
